# Substance use disorders and co-morbidities among Asian Americans and Native Hawaiians/Pacific Islanders

**DOI:** 10.1017/S0033291714001330

**Published:** 2014-06-20

**Authors:** L.-T. Wu, D. G. Blazer

**Affiliations:** 1 Department of Psychiatry and Behavioral Sciences, School of Medicine, Duke University Medical Center, Durham, NC, USA; 2 Center for Child and Family Policy, Duke University, Durham, NC, USA

**Keywords:** Addiction treatment, Asian Americans, co-morbidity, Native Hawaiians, Pacific Islanders, substance use disorder

## Abstract

**Background:**

Asian Americans (AAs) and Native Hawaiians/Pacific Islanders (NHs/PIs) are the fastest growing segments of the US population. However, their population sizes are small, and thus AAs and NHs/PIs are often aggregated into a single racial/ethnic group or omitted from research and health statistics. The groups' substance use disorders (SUDs) and treatment needs have been under-recognized.

**Method:**

We examined recent epidemiological data on the extent of alcohol and drug use disorders and the use of treatment services by AAs and NHs/PIs.

**Results:**

NHs/PIs on average were less educated and had lower levels of household income than AAs. Considered as a single group, AAs and NHs/PIs showed a low prevalence of substance use and disorders. Analyses of survey data that compared AAs and NHs/PIs revealed higher prevalences of substance use (alcohol, drugs), depression and delinquency among NHs than among AAs. Among treatment-seeking patients in mental healthcare settings, NHs/PIs had higher prevalences of DSM-IV diagnoses than AAs (alcohol/drug, mood, adjustment, childhood-onset disruptive or impulse-control disorders), although co-morbidity was common in both groups. AAs and NHs/PIs with an SUD were unlikely to use treatment, especially treatment for alcohol problems, and treatment use tended to be related to involvement with the criminal justice system.

**Conclusions:**

Although available data are limited by small sample sizes of AAs and NHs/PIs, they demonstrate the need to separate AAs and NHs/PIs in health statistics and increase research into substance use and treatment needs for these fast-growing but understudied population groups.

## Introduction

Asian Americans (AAs) include people with origins in the Far East, Southeast Asia and the Indian subcontinent (Asian Indians, Chinese, Filipinos, Japanese, Koreans, Vietnamese, and other Asians). Native Hawaiians and other Pacific Islanders (NHs/PIs) include people having origins in Hawaii, Guam, Samoa, or other Pacific Islands. Data from the 2010 US Census showed that AAs and NHs/PIs were the fastest growing segments of the US population (US Census Bureau, 2011), increasing at least three times faster than the total US population. In 2011, an estimated 18.2 million AAs and 1.4 million NHs/PIs were US residents (US Census Bureau, 2013).

Substance use disorders (SUDs) and treatment needs among AAs and NHs/PIs have been understudied in comparison with other racial/ethnic groups. In the 1990 census, self-designated AAs and NHs/PIs were classified as one group. A 1997 directive from the Office of Management and Budget called for separating AAs and NHs/PIs in census reporting, and in 2000 the census began reporting the groups separately. Epidemiological and clinical studies of substance use and SUDs have often combined the two groups as ‘Asian Americans and Pacific Islanders (AAPI)’ or reported the pooled groups as simply ‘Asians’, which is not optimal for a variety of reasons. At least 20 subgroups make up the 17 million people who designate themselves as AAs and NHs/PIs (SAMHSA, 2010*a*). The statistical power in almost all data sets used to estimate prevalence and correlates of SUDs is not sufficient to disaggregate the individual groups.

The acculturation and enculturation experiences of AAs and NHs/PIs in the dominant Caucasian culture have been vastly different. AAs have arrived in the USA as immigrants (e.g. the arrival of the first Japanese immigrants in 1843 and the role of Chinese workers in building the transcontinental railroad during 1865–1869), and even today, the majority of AAs are foreign-born (SAMHSA, 2010*a*). By contrast, NHs/PIs are natives of their homelands, which have been annexed by the USA and populated by dominant American racial/ethnic groups. Many NHs/PIs move away from their native homes to other geographic locations in the USA. These differences in background between the groups may contribute to different substance use behaviors.

In response to the growing sizes of AAs and NHs/PIs and the need for empirical data to inform research efforts and health policy, we reviewed population and clinical data about substance use, SUDs (alcohol and drugs) and SUD treatment use by these two groups. To provide the context for the review, we described national data on demographics of AAs and NHs/PIs, compared prevalences of alcohol and drug use, and summarized estimates of SUDs. Finally, we considered prevalences of use of alcohol or drug abuse services and psychiatric co-morbidities as they related to treatment needs for AAs and NHs/PIs.

## Method

We searched PubMed and Google Scholar using the key words Asian Americans, Native Hawaiians, Pacific Islanders, substance use, alcohol use, marijuana use, drug use, SUD (alcohol, drugs), substance abuse treatment, and co-morbidity. We excluded case reports, non-empirical (data-based) reports, and publications not in English. We used the related citations link to find additional publications. To reflect recent trends in substance use among AAs and NHs/PIs, we focused on the findings from population-based studies in the past 5 years that had an adequate sample size.

### Demographics of Asian Americans versus Native Hawaiians/Pacific Islanders

AAs are the fastest growing racial/ethnic group in the country (see Table 1). By 2050, AAs are projected to make up about 9% (40.6 million) of the US population (US Census, 2011*b*). Two-thirds of AAs are foreign-born; 57% of these were naturalized US citizens in 2010. Forty-two percent speak English well, yet only 11% speak only English. The vast majority (76%) of AAs live in large metropolitan areas (Wu *et al.* 2013*b*). One half of the AA-alone population aged ⩾25 years have a bachelor's degree or higher level of education (US Census, 2013). AAs resemble Whites in having higher levels of family income than other racial/ethnic groups (DeNavas-Walt *et al.* 2012; Wu *et al.* 2014).

**Table 1.** Demographics of Asian Americans and Native Hawaiians/Pacific Islanders in the USA

	Asian Americans	Native Hawaiians/Pacific Islanders
Population size, 2000 census	11.9 million	874 000
Population size, 2010 census	17.3 million	1.2 million
	5.6% of the US population	0.4% of the US population
Percentage growth: 2000 *v*. 2010	46%	40%
Percentage reporting multiple race	15.3%	55.9%
Geographic distribution	West 46%, South 22%, Northeast 20%, Midwest 12%	West 71%, South 16%, Northeast 7%, Midwest 6%
States with relatively high numbers of the population	California (5.6 million), New York (1.6 million), Texas (1.1 million), New Jersey (0.8 million), Hawaii (0.8 million), Illinois (0.7 million), Washington (0.6 million), Florida (0.6 million), Virginia (0.5 million), Pennsylvania (0.4 million)	Hawaii (356 000), California ( 286 000), Washington (70 000), Texas ( 48 000), Florida (40 000), Utah (37 000), New York (36 000), Nevada (33 000), Oregon (26 000), Arizona (25 000).
Larger subgroups	Chinese (4 million), Filipinos (3.4 million), Asian Indians (3.2 million), Vietnamese (1.7 million), Koreans (1.7 million), Japanese (1.3 million)	Native Hawaiian (527 077), Samoan (184 440), Guamanian or Chamorro (147 798)
Poverty rate	12.8% (for the Asian alone population in 2011)	21.5% (for the Native Hawaiians and Other Pacific Islanders alone population in 2011)
Education	50% (for the Asian alone population aged ⩾25 years who had a bachelor's degree or higher level of education in 2011)	14.5% (for the Native Hawaiians and Other Pacific Islanders alone aged ⩾25 years who had a bachelor's degree or higher in 2011)

Sources of data: US census (2012*a,b*, 2013).

NHs/PIs (1.4 million in 2011) are growing in numbers at 3–4 times the rate of the overall US population (US Census, 2012*b*, 2013). States with relatively high proportions of NHs/PIs are Hawaii, Alaska, Utah, Nevada and Washington (US Census, 2012*a*). Just over half of NHs/PIs live in large metropolitan areas (Wu *et al.* 2013*b*). The NH/PI population (median age: 27.1 years) is younger than the AA population (median age: 33.5 years) and the overall US population (median age: 37.3 years) (US Census, 2013). About 30% speak a language other than English at home (HHS, 2013). In 2011, 14.5% of NHs/PIs aged ⩾25 years had a bachelor's degree or higher level of education (US Census, 2013). In summary, NHs/PIs are less educated and less likely to be foreign-born compared to AAs, and they are more likely to speak English as their only language.

### Alcohol and drug use

Because studies of SUDs typically include a small sample size of AAs and NHs/PIs, research has focused mainly on descriptive prevalences of alcohol and any drug use (see Table 2). AAs and NHs/PIs have either been pooled into an ‘AAPI’ group or combined with other racial/ethnic groups as ‘other’. There is limited information about prevalences of use of illicit drugs among AAs *versus* NHs/PIs (Wu *et al.* 2013*b*,*c*). Earlier studies of AAs show that those who were born in the USA and AAs of mixed heritage had elevated prevalences of alcohol/drug use (Price *et al.* 2002; Wong *et al.* 2004).

**Table 2.** Prevalences of alcohol and illicit/non-medical drug use among Asian Americans (AAs) and Native Hawaiians/Pacific Islanders (NHs/PIs)

Authors/citation	Dataset, Age	Sample size	Substance	Selected findings
SAMHSA, 2011	National sample	3763 AAs	Alcohol and drugs	AA past-month use prevalence
	NSDUH			Alcohol 7.4%, marijuana 2.9%, prescription drugs 1.8%
	12–17 years			Born in the USA
				Alcohol 8.7%, marijuana 3.2%, prescription drugs 1.4%
				Not born in the USA
				Alcohol 4.7%, marijuana 2.1%, prescription drugs 2.7%
				Aged 12–14 years
				Alcohol 2.7%, marijuana 0.5%, prescription drugs 1.6%
				Aged 15–17 years
				Alcohol 11.5%, marijuana 4.9%, prescription drugs 2.0%
Lowry *et al.* 2011	National sample	56 773	Alcohol and drugs	Past-month use prevalence
	YRBSS			AA: alcohol 25.6%, heavy drinking 12.1%, marijuana 9.0%
	High school students, age unavailable			NH/PI: alcohol 44.2%, heavy drinking 32.3%, marijuana 23.0%
				Female AA: alcohol 24.9%, heavy drinking 10.2%, marijuana 9.2%
				Male AA: alcohol 26.2%, heavy drinking 13.8%, marijuana 8.8%
				Female NH/PI: alcohol 45.7%, heavy drinking 31.5%, marijuana 19.5%
				Male NH/PI: alcohol 42.5%, heavy drinking 33.2%, marijuana 26.8%
SAMHSA, 2010*a*	National sample	7629 AAs	Alcohol and drugs	AA past-month use prevalence
				Alcohol 39.8%, binge alcohol 13.2%, illicit or non-medical drugs 3.4%
	NSDUH			Born in the USA
				Alcohol 56.0%, binge alcohol 22.0%, illicit or non-medical drugs 7.3%
	⩾ 18 years			Not born in the USA
				Alcohol 35.9%, binge alcohol 11.1%, illicit or non-medical drugs 2.5%
				Alcohol use by age
				Female: 18–25 years 43.6%, 26–49 years 32.0%, ⩾50 years 18.3%
				Male: 18–25 years 53.5%, 26–49 years 53.6%, ⩾50 years 45.0%
				Illicit or non-medical drug use by age
				Female: 18–25 years 6.9%, 26–49 years 1.4%, ⩾50 years 3.5%
				Male: 18–25 years 11.4%, 26–49 years 3.1%, ⩾50 years 1.0%
Wu *et al.* 2013*b*	National sample	275 685	Drugs	AA: past-year use prevalence in 2011
				Any drug 6.8%, marijuana 4.9%, opioid analgesic 1.8%, inhalant 0.7%, cocaine 0.8%, hallucinogen 1.0%, heroin < 1.0%, stimulant 0.3%, sedative < 1.0%, tranquilizer 0.8%
	NSDUH			
	⩾ 12 years			
				NH/PI: past-year use prevalence in 2011
				Any drug 21.2%%, marijuana 18.8%, opioid analgesic 2.7%, inhalant 2.7%, cocaine 3.2%, hallucinogen 3.8%, heroin 0.8%, stimulant 1.7%, sedative < 1.0%, tranquilizer 1.5%
				Adjusted OR (95% CI) of past-year use
				NH/PI *v*. AA: any drug use 1.70 (1.20–2.40), marijuana use 1.72 (1.12–2.63), heroin use 5.06 (1.25–20.49)
				NH/PI *v*. White: any drug use 1.01 (0.76–1.35), marijuana use 0.87 (0.60–1.27), heroin use 1.63 (0.67–3.99)
				AA *v*. White: any drug use 0.60 (0.52–0.68), marijuana use 0.51 (0.44–0.59), heroin use 0.32 (0.10–1.02)

NSDUH, National Survey on Drug Use and Health; YRBSS, Youth Risk Behavior Surveillance System; OR, odds ratio; CI, confidence interval.

A 2010 report used data from the 2004–2008 National Surveys on Drug Use and Health (NSDUH) to present findings on alcohol and drug use prevalences among AAs aged ⩾18 years (SAMHSA, 2010*a*). AAs had lower past-month prevalences of alcohol and drug use than the national average: alcohol use (39.8% *v*. 55.2%), binge alcohol use (13.2% *v*. 24.5%) and illicit/non-medical drug use (3.4% *v*. 7.9%). Past-month alcohol use was reported by 51.9% of Korean Americans, 48.3% of Japanese Americans, 41.3% of Chinese Americans, 38.7% of Vietnamese Americans, 38.1% of Filipino Americans and 32.1% of Asian Indians. Binge drinking varied from 25.9% among Korean Americans to 8.4% among Chinese Americans (Filipino Americans 15.0%, Japanese Americans 14.5%, Vietnamese Americans 14.0%, and Asian Indians 9.5%). The prevalence of any illicit or non-medical drug use also varied (Korean Americans 5.3%, Japanese Americans 6.2%, Chinese Americans 2.1%, Vietnamese Americans 5.3%, Filipino Americans 3.2%, and Asian Indians 2.1%).

A similar pattern is noted for substance use among adolescents (12–17 years) from the pooled 2004–2009 NSDUH data (SAMHSA, 2011), as shown in Table 2. Prevalence of alcohol use ranged from 9.7% among Filipino Americans to 5.1% among Asian Indians 5.1%. Marijuana use varied from 5.2% among Korean Americans to 1.0% among Asian Indians. Available drug use estimates for NHs/PIs are even more limited, as studies have often pooled NHs/PIs with other racial/ethnic groups. Studies that separated AAs and NHs/PIs in the analysis have demonstrated differences in behavioral and mental health between AAs and NHs/PIs (Wong *et al.* 2004; Kim & McCarthy, 2006; Wu *et al.* 2013*a*,*b*,*c*). For example, Lowry *et al.* (2011) pooled data from 4 years (2001, 2003, 2005, and 2007) of the Youth Risk Behavior Survey to examine behavioral health among high school students (*n* = 56 773). NHs/PIs had higher prevalences than AAs in current substance use (see Table 2), and also in being sexually active, carrying a weapon, engaging in a physical flight, suicidal ideation, and attempting suicide. Higher prevalences of these behavioral health indicators were observed in both genders of NHs/PIs and showed little gender differences. Overall, AAs showed lower prevalences, and NHs/PIs were similar to Whites, Blacks and Hispanics in prevalence of these behavioral health indicators (Lowry *et al.* 2011).

Recently, Wu *et al.* (2013*b*) examined national trends in illicit/non-medical drug use prevalences among AAs and NHs/PIs aged ⩾12 years for 2005–2011 (*n* = 275 685) (see Table 2). Between 2005 and 2011, there were no significant variations in past-year prevalences of drug use among NHs/PIs and AAs. Drug use was much more common among NHs/PIs than AAs and Whites. Controlling for multiple covariates, NHs/PIs showed higher adjusted odds ratios (aORs) than AAs for any drug use [aOR 1.70, 95% confidence interval (CI) 1.20–2.40], marijuana use (aOR 1.72, 95% CI 1.12–2.63) and heroin use (aOR 5.06, 95% CI 1.25–20.49). Adjusted analyses also found that NHs/PIs resembled Whites in odds of use of most drugs.

Regardless of race/ethnicity, age was the most reliable correlate of recent illicit or non-medical drug use among AAs and NHs/PIs, which generally showed an increase in adolescence and young adulthood, then a decline thereafter (Wu *et al.* 2013*b*, 2014). Low household income was associated with elevated odds of drug use among AAs but not among NHs/PIs; other demographics (e.g. gender, urbanicity, residential move) were inconsistent correlates of drug use. However, behavioral and mental health variables (e.g. delinquency, being arrested, tobacco use, alcohol use, major depression) were associated with past-year drug use among both AAs and NHs/PIs (Wu *et al.* 2013*b*, 2014).

### Alcohol and drug use disorders

Data from the 2001–2003 National Comorbidity Survey Replication (NCS-R) showed that about 7.9% of White, 6.3% of Black and 9.1% of Hispanic adults aged ⩾18 years met criteria for having a past-year alcohol use disorder (Breslau *et al.* 2005). However, because of the small sample size, estimates for AAs and NHs/PIs are not available. The 2001–2002 National Epidemiologic Survey on Alcohol and Related Conditions (NESARC) included a total of 1332 AA/NH/PI adults aged ⩾18 years. In the NESARC, the pooled AAs/NHs/PIs had a lower prevalence of past-year alcohol use disorder than other groups (Hasin *et al.* 2007), and there were slighter racial/ethnic differences in the prevalence of past-year drug use disorder (Compton *et al.* 2007), as shown in Table 3.

**Table 3.** Alcohol and drug use disorders among Asian Americans (AAs) and Native Hawaiians/Pacific Islanders (NHs/PIs)

Authors/citation	Dataset, Age	Sample size	Substance	Selected findings
Compton *et al.* 2007	National sample	43 093	Drugs	Lifetime drug use disorder
	NESARC			AA/NH/PI 3.8%, White 11.3%, Black 8.7%, Hispanic 7.2%, Native American 18.4%
	⩾18 years			
Hasin *et al.* 2007	National sample	43 093	Alcohol	Lifetime alcohol use disorder
	NESARC			AA/NH/PI 11.6%, White 34.1%, Black 20.6%, Hispanic 21.0%, Native American 43.0%
	⩾18 years			
Wu *et al.* 2011	National sample	72 561	Alcohol or drugs	Past-year alcohol or drug use disorder
	NSDUH			AA/NH/PI: 3.5% (alcohol 2.1%, any drug 2.0%)
				White: 9.0% (alcohol 6.3%, any drug 5.0%
	12–17 years			Black: 5.0% (alcohol 2.3%, any drug 3.7%)
				Hispanic: 7.7% (alcohol 5.5%, any drug 4.4%)
				Native American: 15.0% (alcohol 9.4%, any drug 8.7%)
				Mixed-race: 9.2% (alcohol 6.3%, any drug 6.4%)
Wu *et al.* 2012	National sample	113 672	Alcohol or drugs	Conditional past-year alcohol or drug use disorder in alcohol or drug users who used emergency-department care in the past year
	NSDUH ⩾18 years	users of emergency department		
				AA/NH/PI: 11.7% (alcohol 10.3%, any drug 13.6%)
				White: 15.2% (alcohol 12.5%, any drug 23.3%
				Black: 18.0% (alcohol 14.4%, any drug 25.7%)
				Hispanic: 17.2% (alcohol 14.6%, any drug 23.4%)
				Native American: 28.7% (alcohol 27.0%, any drug 24.5%)
				Mixed-race: 23.6% (alcohol 21.4%, any drug 22.2%)
Wu *et al.* 2014	National sample	394 400	Marijuana	Past-year marijuana use disorder (conditional prevalence of marijuana use disorders in marijuana users)
	NSDUH ⩾ 12 years			
				AA: 0.8% (17.0% in marijuana users)
				NH/PI: 1.3% (12.6% in marijuana users)
				White: 1.5% (13.7% in marijuana users)
				Black: 2.5% (19.1% in marijuana users)
				Hispanic: 1.8% (19.6% in marijuana users)
				Native American: 3.7% (24.6% in marijuana users)
				Mixed-race: 2.8% (15.8% in marijuana users)

NESARC, National Epidemiologic Survey on Alcohol and Related Conditions; NSDUH, National Survey on Drug Use and Health.

Among adolescents (age 12–17 years), analyses of data from the 2005–2008 NSDUH (*n* = 72 561) showed that the pooled AA/NH/PI group was similar to Blacks in prevalence of past-year SUDs but had lower prevalences than Whites, Hispanics, Native Americans and mixed-race people (Wu *et al.* 2011). Conditional prevalences of alcohol or drug use disorders among alcohol or drug users (the liability to abuse or dependence given use) suggested that the pooled AA/NH/PI group was similar to Blacks in the prevalence of drug use disorder given use (Wu *et al.* 2011).

In an analysis of SUDs by emergency department use in national samples of adults aged ⩾18 years (*n* = 113 672), pooled AAs/NHs/PIs also showed a comparatively low prevalence of SUDs given use (Wu *et al.* 2012). Among past-year alcohol or drug users who received care in an emergency department setting, 11.7% of pooled AAs/NHs/PIs, 15.2% of Whites, 18.0% of Blacks, 17.2% of Hispanics, 28.7% of Native Americans and 23.6% of mixed-race adults met criteria for having an SUD. In controlled analyses, AAs/NHs/PIs did not differ from alcohol-using Whites in the odds of alcohol use disorders but drug-using AAs/NHs/PIs had lower odds of drug use disorders than drug-using Whites.

A more recent study of marijuana use disorder in a national sample of persons aged ⩾12 years separated NHs/PIs and AAs (Wu *et al.* 2014) (see Table 3). In the overall sample, more NHs/PIs than AAs used marijuana in the past year but there were no significant differences in past-year marijuana use disorder in either the total sample (1.3% *v*. 0.8%) or the subsample of marijuana users (12.6% *v*. 17.0%). Controlling for sociodemographic variables, AAs and Hispanics had lower odds of marijuana use than Whites, the odds for Blacks and Native Americans resembled those of Whites, and mixed-race persons had greater odds than Whites of using marijuana. Among marijuana users, AAs had greater odds than Whites of having a marijuana use disorder (aOR 1.88, 95% CI 1.39–2.55), and NH/PI marijuana users were as likely as White users to have a marijuana use disorder. In both AA and NH/PI groups, younger age (especially 12–17 years) and a greater frequency of marijuana use (⩾52 days in a year) were robust correlates of marijuana use disorder.

### Use of alcohol or drug abuse treatment services

#### Surveys of the general populations

Comparisons of national survey data between the 1991–1992 National Longitudinal Alcohol Epidemiologic Survey and the 2001–2002 NESARC showed some declines in the prevalence of any lifetime treatment service use (addiction or mental health programs, non-specialty medical services, mutual aid, human services, or other service) for alcohol problems among adults with a lifetime alcohol use disorder between the years 1991–1992 and 2001–2002 (Whites 20.3% *v*. 14.0%, Blacks 22.2% *v*. 17.1%, Hispanics 19.6% *v*. 16.2% respectively) but results are not available for AAs and NHs/PIs (Chartier & Caetano, 2011).

Findings from the 2001–2002 NESARC indicated that fewer AAs/NHs/PIs with a lifetime alcohol use disorder than other racial/ethnic groups with an alcohol use disorder had ever received any treatment or service for alcohol problems in their lifetime (Cohen *et al.* 2007) (see Table 4).

**Table 4.** Substance abuse service use and co-morbidity among Asian Americans (AAs) and Native Hawaiians/Pacific Islanders (NHs/PIs)

Authors/citation	Dataset, Age	Sample size	Substance	Selected findings
Wu *et al.* 2003	National sample	16 661	Alcohol or drug	Adjusted OR (95% CI) of substance abuse service use in adults with alcohol or drug use problems
	NHSDA ⩾18 years			
				AA/NH/PI *v*. White 0.28 (0.11–0.70), Black *v*. White 0.73 (0.33–1.59), Hispanic *v*. White 0.59 (0.27–1.30), Native American *v*. White 4.74 (1.88–11.98)
Cohen *et al.* 2007	National sample	1716	Alcohol	Any lifetime use of alcohol services in adults with a lifetime alcohol use disorder
	NESARC ⩾18 years			
				AA/NH/PI 9.0%, White 14.0%, Black 17.1%, Hispanic 16.1%, Native American 22.9%
Cummings *et al.* 2011	National sample	12 634	Alcohol or drug	Any past-year use of alcohol or drug services in adolescents with a past-year alcohol or drug use disorder
	NSDUH			
	12–17 years			AA 11.0%, White 11.9%, Black 8.4%, Hispanic 9.8%, Native American 18.2%, Mixed-race 15.6%
Mericle *et al.* 2012	National sample	19 729	Alcohol or drug	Lifetime disorder in adults with a lifetime alcohol use disorder
	CPES ⩾18 years			Depressive disorder: AA 41.6%, White 37.6%, Black 27.5%, Hispanic 29.7%
				
				Anxiety disorders in adults with a lifetime alcohol use disorder: AA 34.4%, White 41.4%, Black 36.1%, Hispanic 31.6%
				Lifetime disorder in adults with a lifetime drug use disorder
				Depressive disorder: AA 47.5%, White 43.9%, Black 33.4%, Hispanic 29.3%
				Anxiety disorders in adults with a lifetime alcohol use disorder: AA 41.1%, White 47.3%, Black 42.5%, Hispanic 35.2%

CPES, Collaborative Psychiatric Epidemiology Surveys; NESARC, National Epidemiologic Survey on Alcohol and Related Conditions; NHSDA, National Household Survey on Drug Use; NSDUH, National Survey on Drug Use and Health; OR, odds ratio; CI, confidence interval.

Any use of treatment services included use of treatment or service at specialty (addiction or mental health) programs, non-specialty medical services, mutual aid, non-specialty human services, or other settings.

In a recent report that analyzed the combined 2003–2011 NSDUH data (SAMHSA, 2013), 4.9% of pooled AAs/NHs/PIs aged ⩾12 years were estimated to need treatment for alcohol (3.9%) or drug (1.4%) abuse (i.e. having a DSM-IV alcohol or drug use disorder or receiving treatment for alcohol or drug use at an addiction or mental health service setting in the past year). Among AAs/NHs/PIs aged 18–25, males and those without health insurance had a higher proportion of needing alcohol or drug abuse treatment services than adolescents and adults older than 25. Among AAs/NHs/PIs with a need for alcohol or abuse treatment services in the past year, 5.3% received alcohol or abuse treatment at a specialty setting (addiction or mental health programs) in the past year. In addition, more AAs/NHs/PIs with a need for illicit drug abuse treatment received specialty treatment (10.9%) than AAs/NHs/PIs with a need for alcohol abuse treatment received specialty treatment (3.7%) (SAMHSA, 2013). These data indicate substantial unmet needs for SUD treatment.

Available but limited national survey data for adolescents suggest few racial/ethnic differences in the prevalence of any substance abuse treatment use among adolescents. Among adolescents aged 12–17 with a past-year alcohol or drug use disorder, Cummings *et al.* (2011) found that more Native Americans than AAs used any substance abuse treatment in the past year. Mixed race individuals fell between these two groups (see Table 4).

#### Clinical samples

Treatment use data from the New York statewide substance abuse treatment and discharge database showed that AAs represented a small proportion (<1%) of the patients in the sample (Yu & Warner, 2013; Yu *et al.* 2014). About 82% of AA patients were male; the mean age of AAs was 34.6 years, which did not differ significantly from Whites, Blacks and Hispanics. Close to 64% of AA patients reported English as their primary language. More AAs had only one treatment episode (71%) in the database than Whites (65%), Blacks (56%) and Hispanics (53%). The data suggested that AAs (43%) may be more likely than Whites (31%), Blacks (39%) and Hispanics (37%) to enter treatment through the criminal justice system (Yu *et al.* 2014).

The Treatment Episode Data Set (TEDS), which collects demographic and substance abuse characteristics of admissions to treatment for abuse of alcohol and/or drugs in facilities that report to individual state administrative data systems, includes substance abuse treatment admission data for pooled AAs/NHs/PIs. AAs/NHs/PIs accounted for 1% of all TEDS admissions, and alcohol was the primary substance involved in 46% of male AA/NH/PI admissions and 38% of female AA/NH/PI admissions. Among male AAs/NHs/PIs, marijuana (21%) and amphetamines/methamphetamines (17%) were more frequently identified than opiates (9%) and cocaine (5%). Among female AAs/NHs/PIs, amphetamines/methamphetamines (23%) and marijuana (19%) were more commonly identified than opiates (13%) and cocaine (5%) (SAMHSA, 2012).

Compared with patterns of substance use in the overall AA/NH/PI admissions, illicit drugs accounted for a high proportion of treatment admissions among young AAs/NHs/PIs (ages 18–25 years) in TEDS. Among these young adults, alcohol was the primary substance involved in 34% of male admissions and 26% of female admissions (SAMHSA, 2010*b*). Methamphetamines and marijuana were the two primary drug classes involved in treatment admissions for AAs/NHs/PIs aged 18–25. The most common source of referrals for young AA/NH/PI admissions was the criminal justice system (male 65%, female 44%), followed by self-referral (male 19%, female 26%) and other community organizations (male 7%, female 17%). Other medical settings accounted for a very low proportion (<7%) of referrals for substance abuse treatment admissions (SAMHSA, 2010*b*).

Collectively, the treatment use data from pooled AAs/NHs/PIs in TEDS suggest high proportions of illicit drug-related admissions among young adults, greater methamphetamine use problems among females, and greater marijuana use problems among males. AAs/NHs/PIs with substance abuse problems may be unlikely to be identified or referred to substance abuse treatment by health-care providers.

SUD prevalences among AAs, NHs/PIs and mixed-race people aged ⩽90 years (*n* = 4572) were examined by Wu *et al.* (2013*a*) in a behavioral health database. DSM-IV diagnoses among patients from 11 hospitals and mental health facilities were systematically captured by an Electronic Health Record system. Overall, 11.3% of AAs aged ⩾12 years had an SUD (3.7% having at least two SUDs). Adolescents aged 12–17 (10.1%) had a higher prevalence of cannabis diagnosis than adults aged 35–49 (10.1% *v*. 2.1%). In the adjusted analysis, AA adolescents and young adults (18–34 years) were two to three times more likely than older adults (⩾50 years) to have an SUD. Overall, 20.1% of NHs/PIs aged ⩾12 years had an SUD (6.5% having at least two SUDs). Adolescents and young adults aged 18–34 had higher prevalences of cannabis diagnosis than NHs/PIs aged ⩾35 years, whereas alcohol, cocaine and polysubstance diagnoses were more prevalent among adults than adolescents. Compared with AAs, NHs/PIs had higher odds of having an SUD (aOR 2.62, 95% CI 2.00–3.42) and multiple SUDs (aOR 2.41, 95% CI 1.55–3.76), even after the analysis was adjusted for age at first treatment, sex, treatment setting, length in psychiatric treatment, and the number of other co-morbid diagnoses. Greater length of psychiatric treatment among NHs/PIs was positively associated with having at least two SUDs. In both groups, males were more likely than females to have an SUD or at least two SUDs (Wu *et al.* 2013*a*). In summary, treatment-seeking NH/PIs showed a higher SUD prevalence than treatment-seeking AAs.

#### Psychiatric co-morbidities

Psychiatric co-morbidities can intensify clinical courses and affect treatment outcomes of individuals with an SUD (Najt *et al.* 2011). However, there are very limited co-morbidity data available for AAs and NHs/PIs. Of the pooled AAs/NHs/PIs (*n* = 1332) in the 2001–2002 NESARC, 7.4% had a past-year mood disorder (similar to Whites, 9.4%), 6.9% had a past-year anxiety disorder (lower than Whites, 11.7%), and 10.1% had a personality disorder (lower than Whites, 14.6%) (Huang *et al.* 2006). In the NESARC, lifetime alcohol use disorder among pooled AAs/NHs/PIs (*n* = 1332) was associated with past-year mood disorder (aOR 3.6, 95% CI 1.4–8.9), anxiety disorder (aOR 3.6, 95% CI 1.4–8.9) and personality disorder (aOR 5.0, 95% CI 2.1–12.0). Because of the small sample size, associations of drug use disorder with other mental disorder are unclear. In another analysis of more recent national samples, past-year drug use among AAs was associated with major depressive episode (aOR 1.85, 95% CI 1.26–2.71) (Wu *et al.* 2013*b*), and past-year marijuana use disorder among AA marijuana users was associated with major depressive episode (aOR 2.11, 95% CI 1.10–4.05) (Wu *et al.* 2014).

Using a pooled sample from the Collaborative Psychiatric Epidemiology Surveys (including the NCS-R, National Survey of American Life, and National Latino and Asian American Study), Mericle *et al.* (2012) compared lifetime co-morbid prevalences of psychiatric disorders among AAs. Co-morbidity was high, as shown in Table 4. AAs with co-morbid SUD and other mental disorders did not differ from Whites, Blacks and Hispanics in the prevalence of prior psychiatric hospitalization (22.8% in AAs), history of attempted suicide (17.2% in AAs) and difficulty in paying bills (57.6% in AAs). However, a higher proportion of AAs with co-morbid SUD and other mental disorders were unemployed compared with Whites (21.1% *v*. 2.5%) (Mericle *et al.* 2012).

Research on electronic health record data of community residents (*n* = 4572) provided clinical profiles of AAs and NHs/PIs who accessed mental or substance abuse care at one of the 11 hospitals and mental health centers (Wu *et al.* 2013*a*). Comparisons between treatment-seeking AAs with an SUD and treatment-seeking AAs without an SUD showed no significant differences in diagnoses for mood (50.0% among AAs with an SUD *v*. 54.7% among AAs without an SUD), anxiety (26.0% *v*. 31.3%), adjustment (14.4% *v*. 22.7%) or personality disorder (14.4% *v*. 8.8%); schizophrenic or psychotic (13.5% *v*. 17.9%); attention-deficit/hyperactivity disorder, conduct disorder, oppositional defiant disorder and disruptive behavior (8.7% *v*. 7.7%); or eating (3.8% *v*. 3.4%), cognitive (1.9% *v*. 2.6%) or impulse-control disorder (1.0% *v*. 1.7%). Comparisons between treatment-seeking NHs/PIs with an SUD and treatment-seeking NHs/PIs without an SUD indicated significant differences in diagnoses for mood (73.6% among NHs/PIs with an SUD *v*. 64.2% among NHs/PIs without an SUD), adjustment (33.6% *v*. 43.6%) and personality disorder (20.2% *v*. 10.9%). Overall, treatment-seeking NHs/PIs had higher prevalences than treatment-seeking AAs of mood disorder (62.2% *v*. 50.2%); adjustment disorder (43.5% *v*. 22.1%); attention-deficit/hyperactivity disorder, conduct disorder, oppositional defiant disorder and disruptive behavior (19.9% *v*. 11.8%); and impulse-control disorder (12.2% *v*. 1.7%) diagnoses.

## Discussion

AAs and NHs/PIs are frequently reported as a single group in research and health statistics. Findings from pooled AAs/NHs/PIs indicate a low prevalence of alcohol/drug use but analyses that disaggregate NHs/PIs from AAs reveal a particularly high prevalence of substance use among NHs/PIs in the general population and treatment-seeking samples. Nationally, an estimated 11% of NHs/PIs aged ⩾12 years had a history of major depressive episodes (7% among AAs), 33% used tobacco in the past year (18% among AAs), 61% used alcohol in the past year (52% among AAs), and 21% used illicit or non-medical drugs in the past year (7% among AAs) (Wu *et al.* 2013*b*,*c*). Overall, NHs/PIs resemble Whites in drug use prevalence whereas AAs have lower odds than Whites of using drugs (marijuana, opioids, cocaine, hallucinogens, stimulants, sedatives, tranquilizers). Thus, health statistics from pooled AAs/NHs/PIs may underestimate intervention needs for NHs/PIs.

Compared with AAs, NHs/PIs on average are younger, less educated and less likely to be foreign-born; have lower family incomes; and have greater behavioral risk indicators. Such characteristics, including younger age, US-born, less education, low family income, and behavioral risk indicators (depression, delinquency, suicidal behaviors), are all associated with substance use (SAMHSA, 2010*a*; Lowry *et al.* 2011; Wu *et al.* 2013*b*,*c*, 2014). These differences may account partly for some differences in substance use prevalences between NHs/PIs and AAs. Available data, although descriptive, demonstrate that NHs/PIs are a vulnerable population that requires research efforts to discern risk and protective factors of substance use and to develop culturally appropriate interventions to address substance use (Edwards *et al.* 2010). Adolescents and young adults in addition to low-income NHs/PIs should be included as targets of primary and secondary prevention programs (Wu *et al.* 2013*b*,*c*, 2014). Marijuana use, accounting for about 89% of all past-year drug use among NHs/PIs, should be the primary focus of drug use intervention (Wu *et al.* 2014).

Across AA subgroups, alcohol use is comparatively prevalent among Korean, Japanese and Chinese Americans whereas drug use is comparatively prevalent among Korean, Japanese and Vietnamese Americans (SAMHSA, 2010*a*). Although AAs use fewer substances than NHs/PIs, the burden of substance use problems is growing, especially among Chinese Americans, the largest AA subgroup, as their population size rises rapidly. Alcohol, marijuana and methamphetamines account for most treatment admissions and can be the focus of research and intervention for AAs and NHs/PIs (SAMHSA, 2010*b*, 2012; Wu *et al.* 2013*a*).

Nationally, an estimated 4.5% of adult AAs/NHs/PIs had a past-year alcohol use disorder and 1.4% had a past-year drug use disorder (Compton *et al.* 2007; Hasin *et al.* 2007). Among adolescent AAs/NHs/PIs (12–17 years), an estimated 2.1% had a past-year alcohol use disorder and 2.0% had a past-year drug use disorder (Wu *et al.* 2011). Males, adolescents and young adults are disproportionally affected by SUDs. More recent data estimate that 0.8% of AAs (⩾12 years) and 1.3% of NHs/PIs nationally have a past-year marijuana use disorder and that AAs have a lower prevalence than the national average prevalence (1.7%) (Wu *et al.* 2014). However, research is needed to provide more reliable estimates of alcohol and drug use disorders specifically for AAs and NHs/PIs.

Although reliable estimates of SUD treatment use among AAs and NHs/PIs are lacking, AAs/NHs/PIs as a group considerably underutilize treatment (Wu *et al.* 2003), especially treatment for alcohol problems (SAMHSA, 2013). An estimated 91% of adult AAs/NHs/PIs in a national sample with a lifetime alcohol use disorder had never used an alcohol abuse treatment service (Cohen *et al.* 2007). Among AAs/NHs/PIs (⩾12 years) with a need for alcohol abuse treatment in the past year, only 3.7% received treatment at a specialty (addiction, mental health) setting in the past year; among AAs/NHs/PIs with a need for drug use treatment, just 10.9% received drug abuse treatment at a specialty setting (SAMHSA, 2013). Because prevalence estimates are defined to include any service use (specialty plus non-specialty settings, non-medical human services, self-help groups) and treatment often requires multiple visits, the gap of unmet needs for treatment is much greater than the estimates. The low prevalence of treatment use is related to a low rate (2%) of perceived need for treatment by AAs/NHs/PIs with substance problems (SAMHSA, 2013).

Additionally, denial, stigma, cost of treatment, lack of knowledge about treatment, language barriers, and cultural attitudes to behavioral health may contribute to low rate of treatment use (Wu & Ringwalt, 2004; Wu *et al.* 2007; Smith *et al.* 2010; Hedden & Gfroerer, 2011). AAs may experience a particularly high level of unmet need for SUD care because culture-relate beliefs about occurrences of behavioral and mental illnesses (‘Karma’) and family-related stigma associated with such conditions (e.g. embarrassment, fear of destroying a family reputation) may delay help-seeking and interfere with engagement in treatment (Li & Seidman, 2010). Cost concerns (lacking insurance coverage), inadequate English language proficiency and a lack of knowledge about SUD treatment options (which are developed from the western culture) may also impede treatment use (Yu *et al.* 2009). AAs may delay entering into treatment until substance use impairs daily functioning or causes legal consequences. Treatment use data suggest that AAs tend to use SUD treatment through the criminal justice system (SAMHSA, 2010*b*; Yu *et al.* 2014). AAs and NHs/PIs may benefit from targeted educational interventions that increase their knowledge about the symptoms of SUDs, multiple consequences and the availability of treatment services.

Personality disorder (10.1%) is the most prevalent DSM-IV disorder among adult AAs/NHs/PIs (⩾18 years) identified by survey data (NESARC), followed by lifetime mood (7.4%), anxiety (6.9%), alcohol (4.5%) and drug use (1.4%) disorders (Huang *et al.* 2006). US-born AAs (⩾18 years) have a higher lifetime prevalence than foreign-born AAs (⩾18 years) of mood (16.6% *v*. 11.8%), anxiety (17.8% *v*. 9.1%), alcohol (24.5% *v*. 7.3%) and drug use (8.3% *v*. 2.3%) disorders (Breslau & Chang, 2006). Half of AAs (⩾18 years) with a lifetime alcohol or drug use disorder in a national sample had a lifetime mood (41.0%) or anxiety (35.3%) disorder (Mericle *et al.* 2012). Co-morbid patterns among AAs with an SUD are generally similar to those of Whites, Blacks and Hispanics with an SUD but such survey data for NHs/PIs are lacking. Survey findings are generally limited to descriptive lifetime prevalences, which are inadequate for estimating intervention needs for recent disorders specifically related to AAs and NHs/PIs.

Mood disorder seems to be the most common DSM-IV diagnosis contributing to use of psychiatric care by AAs and NHs/PIs (Wu *et al.* 2013*a*). Among AAs and NHs/PIs with or without an SUD (aged ⩾12 years), the majority of AAs (50–55%) and NHs/PIs (64–74%) had a mood disorder documented in an electronic behavioral health record database (Wu *et al.* 2013*a*). Treatment-seeking data are consistent with survey findings, showing that NHs/PIs have a higher prevalence of SUDs than AAs and that personality diagnosis is associated with SUD among AAs and NHs/PIs. Moreover, treatment-seeking NHs/PIs have more DSM-IV diagnoses than treatment-seeking AAs (mood disorder; adjustment disorder; attention-deficit/hyperactivity disorder, conduct disorder, oppositional defiant disorder, and disruptive behavior; impulse-control disorder), suggesting worse mental health profiles among NHs/PIs than AAs (Lowry *et al.* 2011; Wu *et al.* 2013*a*).

## Future directions

There is a clear need for proper categorization of AAs and NHs/PIs. The pooled AA and NH/PI group is sometimes described as ‘Asian’ in research reports. Use of ‘Asian’ to encompass AAs and NHs/PIs may misinform research and policy, and should be avoided.

The small sample size of AAs and NHs/PIs in typical surveys makes it challenging to have an adequate sample for an in-depth analysis of health status. There is a need for policy support to invest funding in expanding the groups' sample sizes in research projects. One approach is to oversample AAs and NHs/PIs in a given national sample to obtain a sufficiently large sample for estimating substance use indicators for subgroups of AAs and NHs/PIs.

AAs and NHs/PIs are diverse in their cultures and ethnic groups. To design effective intervention programs, research is needed to elucidate substance use behaviors and attitudes regarding treatment among specific ethnic groups (Iwamoto *et al.* 2012). This can be achieved by focusing on a particular geographic region with a high proportion of AAs (or NHs/PIs) to produce in-depth empirical data from an adequate sample while containing research costs. For example, research efforts can start with large subgroups of AAs, such as Chinese, Filipinos, Asian Indians, Vietnamese, Koreans, and Japanese Americans.

Adolescence and young adulthood are the most reliable correlates of recent substance use whereas other demographics are inconsistent correlates. Additionally, behavioral health problems (use of other substances, delinquency, depression) are closely associated with substance use among AAs and NHs/PIs. This emphasizes the need for prevention programs to start early and target all racial/ethnic groups. AAs and NHs/PIs are vastly under-represented in substance use prevention research (Rehuher *et al.* 2008). There is a need for research to evaluate the effects of universal and secondary prevention programs (school- and family-based programs) for AAs and NHs/PIs.

Cultural factors that may be unique and that function as risk or protective factors for substance use deserve research (Okamoto *et al.* 2009). Acculturation (changes in an individual's original cultural patterns as a result of continuous or direct contact with individuals of a different culture) is associated with increased odds of SUD and mental disorders whereas factors related to enculturation (the degree to which an individual endorses values indigenous to their native culture, or ethnic identity measures) are associated with decreased odds of having a mental disorder (Redfield *et al.* 1936; Blanco *et al.* 2013; Burnett-Zeigler *et al.* 2013). It is important to study how preservation and promotion of one's own cultural values, such as participating in racial/ethnic activities and behaviors, and developing a sense of cultural pride, belonging and attachment to one's racial/ethnic group and identity, protect against substance use and psychopathology (Kim & Omizo, 2010; Blanco *et al.* 2013; Burnett-Zeigler *et al.* 2013).

Individuals of multiple races (mixed-race) are a rising population (US Census Bureau, 2011). Mixed-race persons have higher odds than whites and AAs for past-year drug use, are similar to NHs/PIs in drug use, and have the highest prevalence of lifetime major depressive episodes (18.2%) (Wu *et al.* 2013*b*,*c*). Clinically, mixed-race persons and NHs/PIs also have higher prevalences of SUD and other mental disorders than AAs (Wu *et al.* 2013*a*). Mixed-race persons on average have lower household income and are younger than the White and AA populations. Research is needed to increase the accuracy of classifying multiple-race status, and for analysis of and reporting about this population. Although little is known about mixed-race persons' risk factors for substance use, research could explore how socio-economic status interacts with acculturation and enculturation factors and contributes to substance use and mental health conditions.

AAs/NHs/PIs with substance problems are unlikely to enter treatment unless treatment is triggered by the involvement with the criminal justice system (SAMHSA, 2010*b*, 2013; Masson *et al.* 2013). It seems that only a subset of severe or problematic illicit drug users (e.g. men, young adults, the less educated) use treatment. Individuals with primary alcohol problems and those with internalizing mental disorders (e.g. women, older adults, the educated) may particularly underuse treatment. Besides culture-related stigma, research needs to explore multiple levels of barriers and facilitators to treatment (e.g. language inefficiency, immigration status, preference for treatment mode, and support by providers, peers and family) (Griner & Smith, 2006; Fong & Tsuang, 2007; Yu *et al.* 2009; Cabral & Smith, 2011; Masson *et al.* 2013).

The low usage rate of substance abuse treatment emphasizes the need for incorporating screening of substance use, brief intervention and referral to treatment (SBIRT) programs into primary care to increase early detection and recognition of substance use status, along with timely interventions for substance-related problems (Moyer, 2013; Pilowsky & Wu, 2013; Salvalaggio *et al.* 2013). The Affordable Care Act (ACA) has expanded access to mental and substance abuse services (i.e. essential health benefits) to millions of Americans (Beronio *et al.* 2013). An estimated eight in 10 (1.6 million) of eligible uninsured AAs/NHs/PIs may qualify either for tax credits to purchase coverage in the Health Insurance Marketplace or for Medicaid or the Children's Health Insurance Program (CHIP) (Wendt *et al.* 2014). Community outreach can help uninsured individuals learn about the new health insurance coverage. There is also a need to increase the number of trained bilingual staff and health-care providers with the language or cultural skills to support outreach and enrollment efforts, or to provide integrated treatment (SAMHSA, 2014).

## Conclusions

AAs and NHs/PIs differ in aspects of culture, language, immigration status, key socio-economic characteristics, substance use and mental health status. Research reports have traditionally combined both into one group, which provides inadequate information for informing health policy. NHs/PIs are a small but growing population that faces significant health disparities in multiple indicators. NHs/PIs have been overlooked and understudied in the substance and mental health literature. As shown in the *Healthy People 2010 Final Review* report (National Center for Health Statistics, 2012), NHs/PIs and AAs have the least reliable research data available to evaluate their health status. Their growing population size requires research efforts to produce needed substance use and disorder data that can be used to inform interventions and clinical trials and establish evidenced-based therapies for these ethnically and culturally diverse US populations. Such efforts are necessary for informing the *Healthy People 2020* health objectives.
